# Mid-term Clinical and Radiographic Outcomes of the Actis Total Hip System: A Retrospective Study

**DOI:** 10.7759/cureus.77632

**Published:** 2025-01-18

**Authors:** Yasutaka Masada, Tomonori Tetsunaga, Kazuki Yamada, Takashi Koura, Tomohiro Inoue, Ryuichiro Okuda, Tomoko Tetsunaga, Yusuke Yokoyama, Yuki Okazaki, Toshifumi Ozaki

**Affiliations:** 1 Department of Medical Materials for Musculoskeletal Reconstruction, Faculty of Medicine, Dentistry and Pharmaceutical Sciences, Okayama University, Okayama, JPN; 2 Department of Musculoskeletal Health Promotion, Faculty of Medicine, Dentistry and Pharmaceutical Sciences, Okayama University, Okayama, JPN; 3 Department of Orthopaedic Surgery, Faculty of Medicine, Dentistry and Pharmaceutical Sciences, Okayama University, Okayama, JPN; 4 Department of Orthopaedic Surgery, Okayama University Hospital, Okayama, JPN

**Keywords:** actis, hydroxyapatite, mid-term outcome, spot welds, stem, total hip arthroplasty

## Abstract

Introduction

Implant technology for total hip arthroplasty (THA) was developed to improve hip function and patient satisfaction. Actis (DePuy Synthes, Warsaw, IN, USA) is a short fit-and-fill titanium stem, with a medial-collared and triple-taper (MCTT) geometry, that is fully coated with hydroxyapatite (HA). We evaluated the radiographic and clinical outcomes of the Actis Total Hip System during a mean follow-up of five years.

Patients and methods

We retrospectively analyzed data from 80 patients (14 male and 66 female, mean age: 65 ± 8.4 years) who underwent primary THA using Actis stems (anterolateral approach, 60 hips; posterior approach, 20 hips). Radiographs were obtained postoperatively and at the time of the final examination. Radiographic assessments included the alignment of the femoral stem, spot welds, stress shielding, cortical hypertrophy, subsidence (>2 mm), radiolucent line, pedestal formation, Dorr type, canal fill ratio (CFR), and stem fixation. Clinical evaluation included the Japanese Orthopaedic Association Hip-Disease Evaluation Questionnaire (JHEQ) and Harris Hip Score (HHS).

Results

The mean follow-up period was 64.0 ± 6.0 months. No significant differences were observed in the alignment of the femoral components between approaches. Of the 80 hips, 53 (66.3%) showed radiographic signs of stem osseointegration, predominantly in the mid-distal region of the stem at the final follow-up. Multiple logistic regression analysis revealed that younger age and a higher CFR (20 mm proximal to the lesser trochanter) were associated with the presence of spot welds. Mild stress shielding occurred in 25 hips (31.3%), and no patient experienced severe stress shielding. All stems were fixed by bone on growth. The JHEQ and HHS significantly improved at the final assessment.

Conclusion

At the five-year follow-up, patients who received the Actis Total Hip System during THA had good radiographic and clinical outcomes.

## Introduction

Total hip arthroplasty (THA) is a successful surgery that relieves pain in patients with hip diseases, including primary/secondary hip osteoarthritis (OA) and osteonecrosis of the femoral head (ONFH) and improves their quality of life. THA implant technology has evolved for both the acetabular and femoral components, which have been developed to minimize pain, prevent periprosthetic fractures, permit activity, prevent loosening, and increase implant survivorship [1]. The number of THA procedures has been increasing and is predicted to rise to 635,000 surgeries by 2023 in the United States [2], and the use of cementless stems has also been increasing [3,4].

The Corail, which is one of the most widely used hydroxyapatite (HA)-coated femoral stems, has higher survival rates and fewer complications than other femoral stem designs [5]. The Actis Total Hip System (DePuy Synthes, Warsaw, IN, USA) is a short fit-and-fill HA-coated, medial-collared, triple-tapered (MCTT) stem. Actis was commercially launched in Japan in 2018. The widespread use of minimally invasive surgery (MIS), which preserves muscle and soft tissue, has enabled a lower dislocation rate, faster functional recovery, and shorter hospital stays [6,7]. The Actis Total Hip System, owing to a shorter range of stem lengths than the Corail, enables surgeons to insert stems more easily during MIS.

The Actis has good short-term radiographic outcomes for up to one year after surgery [8]. Hunter et al. reported clinically excellent stem survivorship and very low complication rates at the five-year postoperative follow-up [9]. However, the medium-term radiographic outcomes of the Actis Total Hip System remain unknown. This study aimed to report the midterm radiographic and clinical outcomes of the Actis Total Hip System.

## Materials and methods

Study design and participants

**Figure 1:**
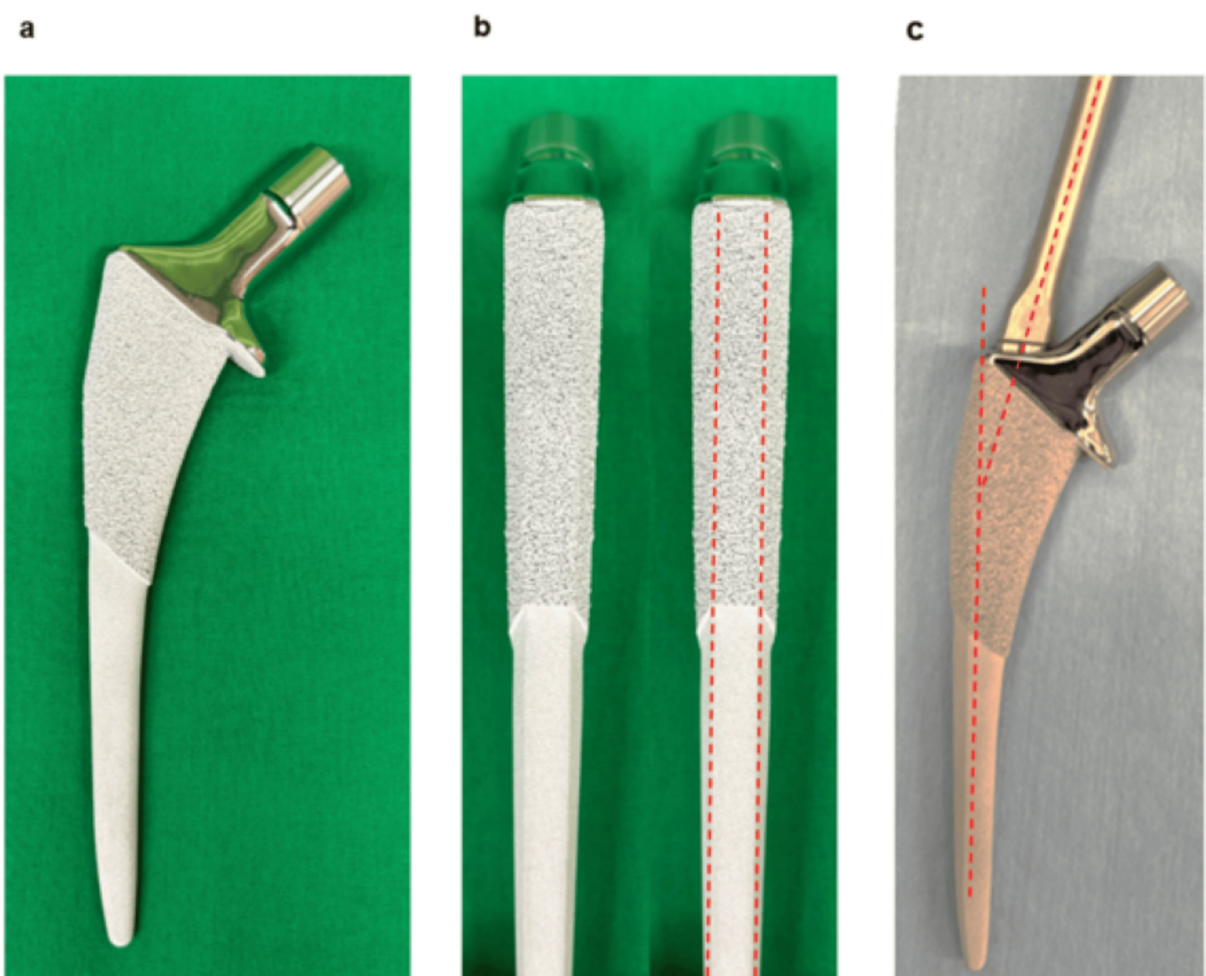
Actis Total Hip System (DePuy Synthes) (a) Medial collared, coated with hydroxyapatite, and lateral shoulder reduced; (b) Tapered from proximal to distal in the anterior/posterior plane, medial/lateral plane, and lateral to medial in the transverse or axial plane; (c) Medial guided inserter tilts at 12° medial against the stem axis to insert the stem smoothly in minimally invasive surgery

This study was approved by the University’s Health Science Institutional Review Board (approval number 2501-055). All procedures were performed following the ethical standards of the institutional and/or national research committee, the 1964 Declaration of Helsinki and its later amendments, or comparable ethical standards. Between April 2018 and June 2020, 85 patients (96 hips) underwent primary THA using Actis at our institution (11 patients underwent staged bilateral THA) (Figure 1). After excluding cases of death (10 patients) and interrupted outpatient visits (six patients), we retrospectively analyzed data from 80 hips (14 male and 66 female). The participants’ characteristics and details are shown in Table 1. The mean age of patients at the time of THA was 65 ± 8.4 years (range, 46-84 years old). The mean follow-up duration was 64.0 ± 6.0 months (range, 53-76 months). The patients’ mean height was 155 ± 7.7 cm (range, 138-173 cm), mean weight was 58 ± 13 kg (range, 40-105.4 kg), and mean body mass index (BMI) was 25 ± 4.2 kg/m² (range, 16.4-37.3 kg/m²). The preoperative diagnoses included OA (66 hips, or 82.5%), ONFH (11 hips, or 13.8%), rapidly destructive coxarthropathy (RDC) (two hips, or 2.5%), and rheumatoid arthritis (RA) (one hip, or 1.3%). In the OA cases (66 hips), 48 hips were classified as Crowe type I, 11 hips as Crowe type II, five hips as Crowe type III, and two hips as Crowe type IV [10]. The mean canal flare index (CFI) was 3.6 ± 0.7 [11]. We used the Dorr classification of preoperative plain hip joint radiographs to assess and classify femoral bone quality into types A, B, and C [11]. Dorr type A included seven hips, type B included 61 hips, and type C included 12 hips.

**Table 1:** Patient demographics Age, follow-up duration, height, weight, and BMI expressed as mean ± standard deviation and range BMI: body mass index

Parameters	n = 80
Gender (%)	
Male	14 (17.5%)
Female	66 (82.5%)
Age (years)	65 ± 8.3 (46-84)
Follow-up duration (months)	64 ± 5.8 (53-76)
Height (cm)	155 ± 7.7 (138-173)
Weight (kg)	58.5 ± 13.4 (40-105.4)
BMI (kg/m^2^)	25 ± 4.2 (16.4-37.3)
Diagnosis (%)	
Osteoarthritis	66 (82.5%)
Osteonecorosis of femoral head	11 (13.8%)
Rapidly destructive cox arthropathy	2 (2.5%)
Rheumatoid arthritis	1 (1.3%)

Surgical procedure

**Figure 2:**
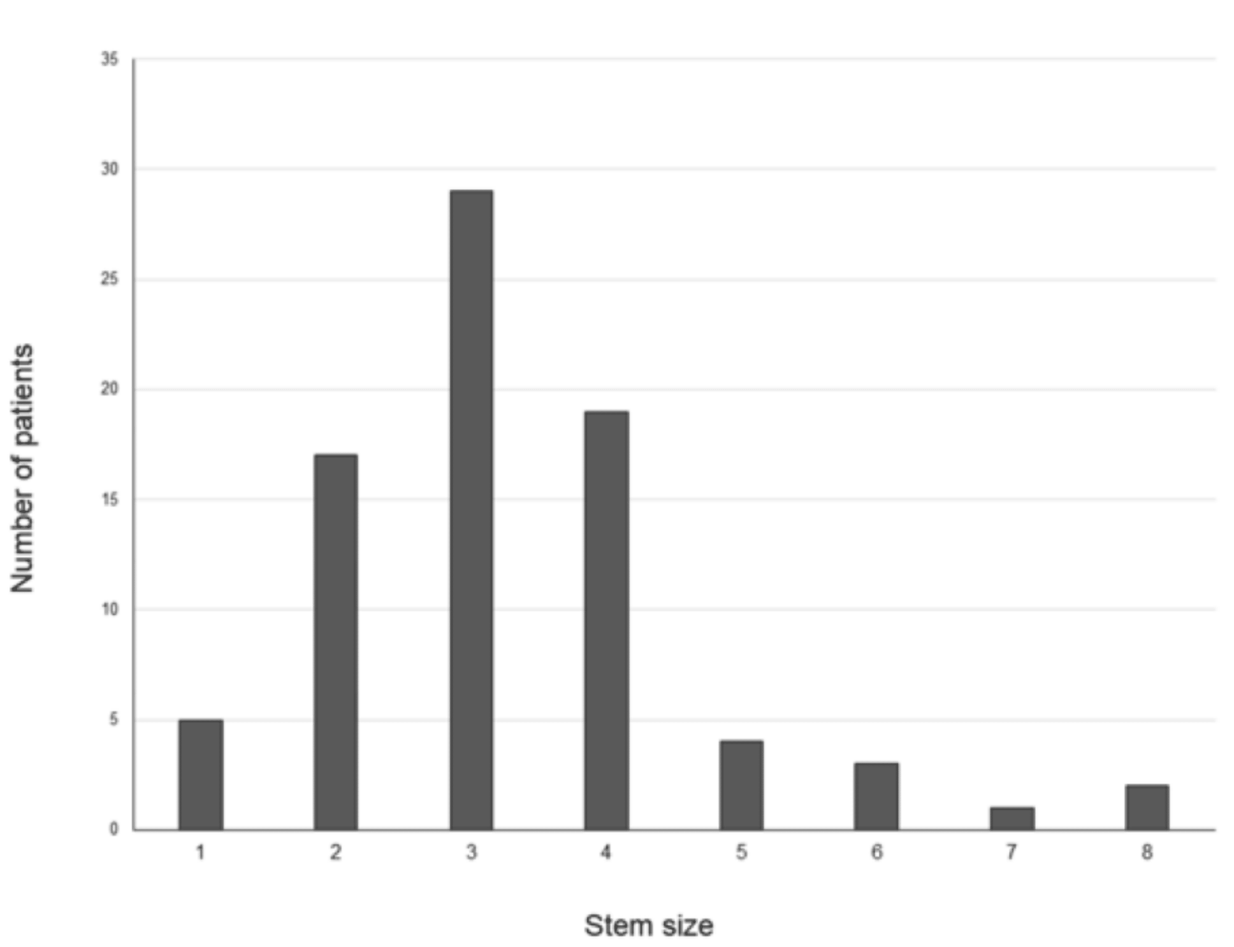
Number of each stem size

All THA procedures were performed by a single surgeon (TT). All surgeries were performed with the patient in the lateral decubitus position. Surgical approaches were selected as either an anterolateral or posterior approach by the surgeons, depending on the degree of each patient’s pelvic deformity, joint contracture, and leg length discrepancy (anterolateral approach, 60 hips; posterior approach, 20 hips). Surgery was performed with a skin incision of approximately 10 cm on the lateral or posterolateral side of the hip. The acetabulum was sequentially reamed using dedicated hemispherical reamers until blood oozed evenly from the bone surface. The PINNACLE® Acetabular Cup System (DePuy Synthes) was used in all surgeries, except for one case that needed KT Plate augmentation (Kyocera Medical, Osaka, Japan). The PINNACLE cup was fixed with two or three screws. Stem size was determined using preoperative 2D or 3D templates. Hand rasping was performed on the planned stem size. When the planned size of the stem trial sank below the osteotomy line, the extra bones were cut with a calcar reamer. The number of implanted components of each size is shown in Figure 2. The mean stem size was 3.3 ± 1.4 (range, 1-8). After the implant was placed, the joint capsule was repaired as much as possible. The day after the surgery, all patients were allowed to walk with full weight bearing.

Radiographic evaluation

Radiographs were obtained postoperatively and at the time of the final examination using an X-ray imaging system (MRAD-A80S RADREX; Canon, Tokyo, Japan). Radiographs of the hip were taken with a wavelength range of 60-80 kV. We assessed spot welds, stress shielding, cortical hypertrophy, subsidence, radiolucent lines, pedestal formation, and stem fixation by using anteroposterior radiographs. The spot welds were recorded based on the Gruen zonal distribution [12]. Stress shielding was defined according to Engh’s classification (zero to second degree was defined as mild, and third to fourth degree was defined as severe) [13]. Subsidence was measured using a perpendicular line drawn from the greater trochanter to the lateral border of the implant, and from the collar to the lesser trochanter as a reference [14]. A difference of ≥2 mm between these measurements was considered important. The alignment of the femoral stem was based on the axial alignment of the proximal femur on the anteroposterior radiographs. Cases in which the stem was inserted in ≥2° of varus or valgus were classified as varus and valgus, respectively. On the lateral radiograph, stem insertion was classified as neutral, flexion, or extension alignments. Stem fixation was evaluated by using the classification of biological fixation proposed by Engh et al. to classify bone ongrowth, stable fibrous fixation, and unstable fibrous fixation [15]. We calculated the canal fill ratio (CFR) at three locations: 20 mm proximal to the lesser trochanter, 20 mm distal to the lesser trochanter, and distal to the stem (20 mm proximal to the stem tip) [16,17].

Clinical evaluation

Clinical evaluation of pain and function was performed using the Japanese Orthopaedic Association Hip Disease Evaluation Questionnaire (JHEQ) [18] and the Harris Hip Score (HHS) [19]. The JHEQ was developed to ascertain patient-reported outcome measures (PROMs) and the condition of patients with hip disorders. The original version of the JHEQ was used in this study. The JHEQ consists of pain (28 points), movement (28 points), and mental health (28 points) subscales, with higher scores indicating better outcomes (a total of 84 points). The visual analog scale (VAS) is an evaluation item in the JHEQ. The VAS includes dissatisfaction with the current condition and hip pain in the JHEQ. These were marked from 0 (complete satisfaction or no pain) to 100 (complete dissatisfaction or maximum pain). The HHS was developed to assess the results of hip surgery and has been used to evaluate various hip disabilities, such as osteoarthritis or femoral neck fracture [19]. The original version of the HHS was published in 1969. The original HHS consists of pain (44 points), function (47 points), absence of deformity (4 points), and range of motion (5 points), with a maximum score of 100 points. Lower scores indicate greater disability. We recorded the JHEQ and original HHS scores preoperatively and at the final assessment.

Statistical analysis

Values are presented as the mean ± standard deviation (SD). The factors affecting spot welds and stress shielding were examined using univariate analysis (Student’s t-test or Chi-square test). Age, BMI, CFI, and CFR were compared using the Student’s t-test. Gender, Dorr classification, and stem alignment ratios were compared using the Chi-square test. Differences were considered statistically significant at p < 0.05. Factors that showed significant differences as explanatory variables in the univariate analysis were included in the multiple logistic regression analysis. The JHEQ scores and the HHS scores at the preoperative and final assessments were compared using the Student’s t-test. Statistical analysis was performed using IBM SPSS Statistics for Windows, Version 25 (Released 2017; IBM Corp., Armonk, NY, USA).

## Results

Regarding the alignment of the femoral stem on anteroposterior radiographs, 67 hips (83.8%) were classified as having neutral alignment, six (7.5%) as having varus alignment, and seven (8.8%) as having valgus alignment. In the hips operated on using the anterolateral approach (60 hips), 51 (85.0%) had neutral alignment, three (5.0%) had varus alignment, and six (10.0%) had valgus alignment. In the hips operated on using the posterior approach (20 hips), 16 (80.0%) had neutral alignment, three (15.0%) had varus alignment, and one (5.0%) had valgus alignment. There was no significant difference between the respective approaches in the anteroposterior radiographs (p = 0.2903). On lateral radiographs, 68 hips (85.0%) were classified as having neutral alignment, and 12 (15.0%) as having flexion alignment. In the hips operated on using the anterolateral approach (60 hips), 49 (81.7%) had neutral alignment and 11 (18.3%) had flexion alignment. In the hips operated on using the posterior approach (20 hips), 19 (95.0%) had neutral alignment, and one hip (5.0%) had flexion alignment. Similar to the anteroposterior radiographs, no significant difference was observed between the respective approaches on the lateral radiographs (p = 0.1481).

**Figure 3:**
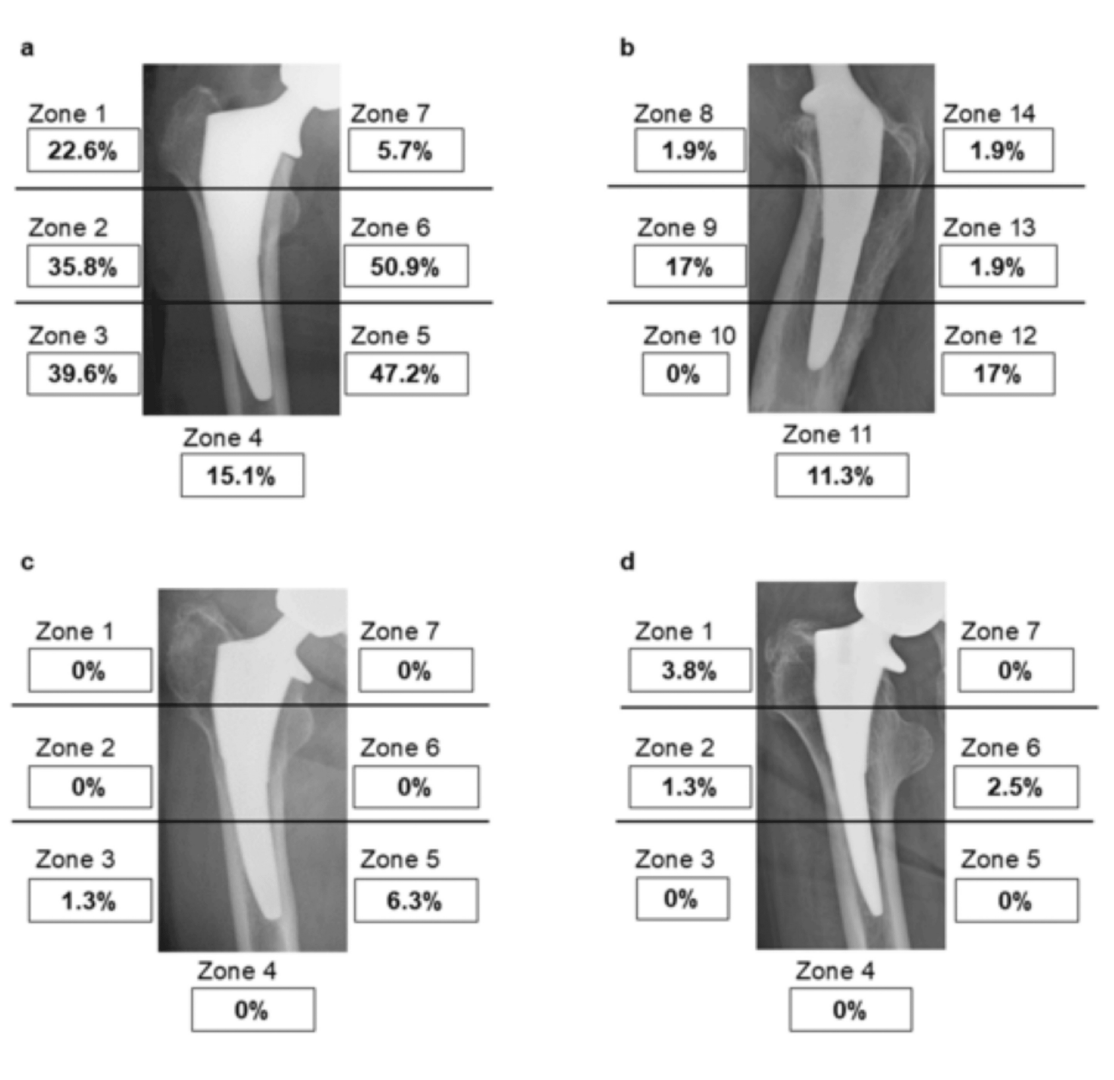
Radiographic outcomes (a) Coronal plane; (b) Sagittal plane; (c) Cortical hypertrophy was observed in Zones 3 and 5; (d) Radiolucent lines were observed in Zones 1, 2, and 6

Spot welds were detected on anteroposterior radiographs in 53 hips (66.3%), particularly in the mid-distal region of the stem (Zones 2-6; Figure 3a). On lateral radiographs, spot welds were detected in 22 hips (27.5%), particularly in Zones 9, 11, and 12 (Figure 3b). Mild stress shielding was observed in 25 hips (31.3%; Grade 1, 17 hips; Grade 2, eight hips), with no hip demonstrating severe stress shielding. Cortical hypertrophy was detected in five hips (6.3%; Zone 3, one hip; Zone 5, five hips; Figure 3c). Subsidence greater than 2 mm was observed in one hip (1.3%). A radiolucent line was observed in four hips (5%; Figure 3d). Pedestal formation was detected in five hips (6.3%). Canal filling is shown in Table 2. The average CFR was 70.2 ± 9.2% at a point 20 mm proximal to the lesser trochanter, 72.4 ± 8.2% at a point 20 mm distal to the lesser trochanter, and 66.7 ± 10.2% at the distal point of the stem. In all cases, stem fixation was achieved via bone ongrowth.

**Table 2:** Average CFR (%) CFR: canal fill ratio

Measurement locations	CFR (%)
20 mm proximal of the lesser trochanter	70.2 ± 9.2
20 mm distal of the lesser trochanter	72.4 ± 8.2
Distal of the stem	66.7 ± 10.2

The results of the univariate analysis for the presence or absence of spot welds showed that younger age (63.2 years vs. 68.2 years, p = 0.005), CFR 20 mm proximal to the lesser trochanter (73.1% vs. 64.5%, p < 0.001), and CFR 20 mm distal to the lesser trochanter (74.5% vs. 68.6%, p < 0.001) were significantly higher in cases with spot welds (Table 3). Multiple regression analysis was performed to investigate the associations of the presence or absence of spot welds, based on the presence of spot welds as the response variable, with age, CFR 20 mm proximal to the lesser trochanter, and CFR 20 mm distal to the lesser trochanter, which were used as explanatory variables. Younger age (p < 0.001; odds ratio, 0.848) and a higher CFR 20 mm proximal to the lesser trochanter (p < 0.001; odds ratio, 1.120) were factors that were significantly associated with the presence of spot welds (Table 4).

**Table 3:** Univariate analysis with presence or absence of spot welds Differences are considered significant for values of p < 0.05 ^a^Chi-square test; ^b^Student t-test BMI: body mass index; CFI: canal flare index; CFR: canal fill ratio; AP: anterior-posterior; ML: medial-lateral

	Spot welds (+)	Spot welds (-)	Test statistics	p-value
	n = 53	n = 27		
Gender (male/female)^a^	9/44	5/22	x^2^ = 0.029	0.86
Age (years)^b^	63.2 ± 8.4	68.2 ± 7.3	t = 2.6	0.005
BMI (kg/m^2^)^b^	24.1 ± 3.9	25.4 ± 4.7	t = 1.36	0.089
CFI^b^	3.7 ± 0.6	3.6 ± 0.8	t = -0.56	0.288
CFR^b^				
Proximal of the lesser trochanter	73.1 ± 8.9	64.5 ± 6.7	t = -4.42	<0.001
Distal of the lesser trochanter	74.5 ± 7.4	68.6 ± 8.6	t = -3.22	<0.001
Distal of the stem	67.9 ± 9.1	64.5 ± 11.8	t = 1.45	0.07
Dorr classification (%)^a^				
A	5 (9.4%)	2 (7.4%)	x^2^ = 0.82	0.66
B	42 (79.2%)	20 (74.1%)		
C	6 (11.3%)	5 (18.5%)		
Alignment (AP)^a^	42/4/7	25/2/0	x^2^ = 3.95	0.139
Neutral/Varus/Valgus				
Alignment (ML)^a^	48/5/0	20/7/0	x^2^ = 3.81	0.051
Neutral/Flexion/Extension				

**Table 4:** Multiple logistic regression analysis with presence or absence of spot welds CFR: canal fill ratio; CI: confidence interval

Variables	Partial regression coefficient	Standard error	Odds ratio	95% CI		p-value
				Lower	Upper	
Age	-0.11	0.03	0.848	-0.17	-0.05	<0.001
CFR (20 mm proximal of the lesser trochanter)	0.11	0.05	1.12	0.02	0.19	<0.001

The JHEQ score improved from 27.8 ± 13.6 at the preoperative assessment to 65.4 ± 12.5 at the final follow-up (p < 0.001). The VAS of dissatisfaction with the hip condition showed improvement, from 76.2 ± 20.2 at the preoperative assessment to 5.2 ± 9.1 at the final follow-up (p < 0.001). Furthermore, the VAS of hip pain improved from 59.4 ± 25.4 at the preoperative assessment to 5.7 ± 9.4 at the final follow-up (p < 0.001). The HHS improved from 53.3 ± 14.4 at the preoperative assessment to 92.9 ± 6.8 at the final follow-up (p < 0.001). No dislocations or periprosthetic fractures (intraoperative or postoperative) were observed during the observation period. One patient required revision surgery due to infection. The overall survival rate was 98.8%.

## Discussion

This study investigated the five-year clinical and radiographic outcomes of patients who underwent THA with the Actis Total Hip System (Figure 1). The survival rate of patients with the Actis Total Hip System was 98.8% five years after surgery. All stems were stable, without any signs of severe stress shielding (Grade ≥3), loosening, or periprosthetic fracture. Spot welds were observed in 66.3% of all hips, particularly in the mid-distal zones (Zones 2, 3, 5, and 6). Multiple logistic regression analysis showed that a higher CFR proximal to the lesser trochanter was a significant factor for the presence of spot welds. The five-year assessment of the Actis Total Hip System showed excellent radiographic and clinical outcomes.

The Actis is a “fit and fill” titanium, MCTT cementless stem, which is designed to optimize bone preservation, spare soft tissue, ensure better stem fit, confer initial stability, and resist early subsidence. The short-term outcomes of the Actis Total Hip System have been reported. In a retrospective cohort study, Actis stems showed a statistically lower incidence of all-cause revision than other stems [20]. The incidence of revision surgery three years after THA was 1.08% (0.43-2.72%) for the Actis stem, whereas it was 2.63% (2.19-3.16%) for other implants. The Actis stem was statistically associated with a 57% lower risk of revisions compared with other implants. Hunter et al. reported excellent survivorship (99.6%) and very low complication rates for the Actis stem at the five-year postoperative follow-up [9]. The use of the MCTT hip constitutes a potential strategy to reduce healthcare resource utilization and costs, helping healthcare payers and decision-makers manage the increasing costs of THA [21].

The fit-and-fill stem provides a strong initial fixation by raising the CFR from the metaphysis to the diaphysis. However, because of its straight and relatively long shape, the rasping technique requires consideration, depending on the approach. A tapered wedge stem that reduced the lateral shoulder of the stem was designed specifically for MIS in THA. However, in a systematic review, single-wedge and double-wedge (fit-and-fill) femoral implants were associated with a three-fold increase in periprosthetic fracture rates [22]. Kaszuba et al. reported no intraoperative calcar fractures, rectus muscle tears, or periprosthetic fractures of the Actis [8]. The medial collar contributes to improved rotational and vertical stem stabilities [23]. Compared to the standard collarless Corail stem, the standard collared Corail stem performed better with regard to any stem revision, including periprosthetic fracture as the endpoint [24]. Although there was no significant difference between the radiographic outcomes of collarless and collared Corail, Giovanoulis et al. demonstrated that subsidence was reduced with the medial collar [23]. We consider the role of the medial collar as “insurance” in the initial fixation of the stem or, in other words, a “safety belt.”

D'Ambrosio et al. reported that femurs with insufficient proximal filling tended to have less favorable radiological outcomes after uncemented THA using a fully HA-coated double-tapered femoral component [17]. Our study showed that a high CFR of the Actis stem is important for good initial fixation and the presence of spot welds. In general, Corail has been called a “silent” stem, which is less likely to accrue peri-stem radiographic changes, such as spot welds, cortical hypertrophy, and stress shielding [25]. However, Liu et al. reported radiographic changes (mild stress shielding, 59.1%; severe stress shielding, 11.1%) in the five-year outcomes of Corail [26]. When using the Corail for narrow canal cases, such as Dorr type A, distal reaming is occasionally required to avoid distal fixation of the stem. However, Actis offers a shorter range of stem lengths than Corail (95-115 mm vs. 93-138 mm). Concerns exist that distal fixation in cases with a narrow canal may result in severe stress shielding during long-term follow-up. Although approximately 10% of the cases in this study were Dorr type A, this short-fit-and-fill system achieved an appropriate fit.

In this study, more spot welds were observed on the anteroposterior radiographs (66.3%) than on the lateral radiographs (27.5%). These results indicated that Actis has the possibility of not being a “silent” stem, such as Corail. Actis introduced hybrid broaching, which consists of compaction (anterior-posterior) and extraction (medial-lateral) to preserve the cancellous bone. We believe that fitting and filling concepts, by excavating the medial and lateral cancellous bones, induced more spot welds on anteroposterior radiographs. Conversely, lateral radiographs showed a “silent” bone reaction, like that of Corail, with compaction of the cancellous bone, except for the HA-induced spot welds where the stem contacts with the femur (Zones 9, 11, 12).

Although the Actis Total Hip System has a very good initial fixation, there are some concerns about radiographic changes in the distal part of the stem. Kaszuba et al. reviewed one-year outcomes of Actis stems and reported the presence of pedestal formation (4.17%), spot welds (90.3%), and cortical hypertrophy (14.6%) [8]. In our five-year radiographic evaluation, there were minor occurrences of pedestal formation, cortical hypertrophy, and severe stress shielding that were comparable to those reported in a previous study. Although distal bone reactions due to HA were detected, spot welds were observed in the proximal part of the Actis stem. Therefore, we concluded that the fixation of the Actis was not distal.

This study had some limitations. First, the sample comprised a small number of patients. The surgical approach in this series was 75% anterolateral and 25% posterior. No significant difference in radiographic findings was observed between the two approaches. However, femoral stems tend to be inserted in a flexed position with an anterolateral approach. In general, when THA is performed using an anterior approach, the stem is more likely to be inserted in a flexed position than when using a posterior approach [27,28]. We consider that, as the number of cases increases, significant differences could be observed in radiographic findings, such as spot welds, if a larger number of patients were studied. Second, all the surgeries were performed by a single surgeon. It is possible that the outcomes of this study are dependent on the surgeon’s skill. Therefore, outcomes from multiple surgeons should be collected to assess whether the outcomes are operator-dependent. Third, this study predominantly involved OA patients, with only a small number of ONFH, RDC, and RA patients. The radiographic findings could change if the proportion of RDC [29] and RA [30] patients, which are characterized by osteoporotic bone, increases. However, no comparative study by disease was conducted in this study due to the small number of RDC and RA patients. This is one of the limitations of this study. Finally, this study was performed using radiography. Computed tomography (CT) evaluation is preferred for a more detailed analysis and to reduce inter-rater variability. Previous reports of radiographically evaluated MCTT stems included only short-term outcomes [8,20]. Our study of the five-year outcomes of Actis showed excellent radiographic and clinical outcomes. The long-term outcomes of Actis should be further investigated in studies designed to overcome the aforementioned limitations.

## Conclusions

We investigated the midterm radiographic and clinical outcomes of patients who received Actis-based THA. Although further long-term observations are needed, the five-year radiographic and clinical outcomes of the Actis Total Hip System are excellent. Younger age and a higher CFR at the proximal lesser trochanter are associated with osseointegration.
